# Attitudes and perceptions towards hypoglycaemia in patients with diabetes mellitus: A multinational cross-sectional study

**DOI:** 10.1371/journal.pone.0222275

**Published:** 2019-10-24

**Authors:** Abdallah Y. Naser, Ian C. K. Wong, Cate Whittlesea, Hassan Alwafi, Amjad Abuirmeileh, Zahra Khalil Alsairafi, Fawaz Mohammad Turkistani, Nedaa Saud Bokhari, Maedeh Y. Beykloo, Dalal Al-Taweel, Mai B. Almane, Li Wei

**Affiliations:** 1 Research Department of Practice and Policy, UCL School of Pharmacy, London, the UK; 2 Isra University, Faculty of Pharmacy, Amman, Jordan; 3 Centre for Safe Medication Practice and Research, Department of Pharmacology and Pharmacy, The University of Hong Kong, Hong Kong; 4 Department of Pharmacy Practice, Kuwait University, Kuwait, Kuwait; 5 Alnoor hospital, Ministry of Health, Mecca, Saudi Arabia; 6 Sabah Al-Ahmad Cardiology Center Pharmacy, Al Amiri Hospital, Kuwait, Kuwait; Tabriz University of Medical Sciences, IR Iran, ISLAMIC REPUBLIC OF IRAN

## Abstract

**Background:**

Preventing hypoglycaemia is an essential component of diabetes self-management that is affected by patients’ attitudes and perceptions. This study aimed to explore the hypoglycaemia problem-solving ability of patients who have diabetes mellitus and factors that determine their attitudes and perceptions towards their previous events.

**Methodology:**

A cross-sectional study was conducted between October 2017 and May 2018 in three Arab countries (Jordan, Saudi Arabia and Kuwait) in patients with diabetes mellitus, who were prescribed antidiabetic therapy and had experienced hypoglycaemic events in the past six months. The Hypoglycaemia Problem-Solving Scale was used in this study. This scale contains two subscales, problem orientation (six questions) and problem-solving skills (eighteen questions), using a five-point Likert scale (range 0–4). Multiple linear regression analysis was used to identify predictors of hypoglycaemia problem-solving abilities.

**Results:**

A total of 895 patients participated in this study from the three countries (300 in Jordan, 302 in Saudi Arabia, and 293 in Kuwait). The average age of the patients was 53.5 years (standard deviation = 13.7) and 52.4% (n = 469) were males. Patients had moderate overall problem-solving ability with a median score of 63.00 (interquartile range = 13.00). Patients’ problem-solving skills score (68.1%) was better than their problem-orientation skills score (58.3%). The highest sub-scale scores were for detection control, setting problem-solving goals, and evaluating strategies, 75.0%. The lowest sub-scale score was for problem-solving perception and immediate management, 50.0%. Older age, being educated, being married, having T2DM, prescribed insulin therapy, and not having been admitted to hospital for hypoglycaemia were important predictors of patients’ problem-solving ability (p < 0.05).

**Conclusions:**

Healthcare professionals are advised to provide more education to patients on how to self-manage hypoglycaemic events. Specifically, they should focus on the overall problem-solving perception of hypoglycaemia and its immediate management.

## Introduction

Antidiabetic medications are essential for the management of patients with type 1 Diabetes Mellitus (DM), and for type 2 DM that is not controlled after appropriate lifestyle modifications. All medications can be associated with adverse events [1] and one of the most commonly encountered adverse events among patients with DM is hypoglycaemia [2]. A previous history of hypoglycaemia is considered a risk factor for recurrent hypoglycaemic events [3]. However, hypoglycaemia is considered an avoidable adverse event and recurrent episodes can be prevented by improving patients’ self-management skills, including healthy diet, exercise, self-monitoring of glucose, taking medications regularly, healthy coping, reducing risk behaviours, and problem solving [4–6].

Problem-solving ability is a cognitive behavioural process in which a person develops strategic thinking techniques to solve specific problems faced in everyday activities [7]. A previous systematic review reported that the problem-solving abilities of patients with diabetes are important and form an essential part in the self-management of their disease [8]. These problem-solving abilities also play a critical role in achieving better control over hypoglycaemic and hyperglycaemic events [9]. In addition, patients’ knowledge of and attitude towards their disease play an important role in the success of diabetes management and prevention of disease development and complications [10]. Therefore, it is important to explore the attitudes and perceptions of patients with diabetes towards their hypoglycaemic events in order to be able to determine their ability to manage any hypoglycaemic events that may occur during their lifetime. In patients with diabetes, measuring problem-solving abilities linked to hypoglycaemic events is vital as it assesses their ability to manage any unpredictable change in their blood glucose level, leading to better disease control [5, 11]. The Hypoglycaemia Problem-Solving Scale (HPSS) is a validated tool designed to assess patients’ hypoglycaemia problem-solving ability [5]. The original HPSS was developed and examined for its psychometric properties in 313 patients in Taiwan with type 1 and type 2 DM aged 20 years and above [5]. The original questionnaire was tested in terms of its face, content and divergent validity.

DM is a prevalent disease in the Middle East region and particularly in Arab countries [12]. However, there is no study that assesses the problem-solving ability of patients in the Middle East region in the context of hypoglycaemic events. The primary aim of this study was to assess hypoglycaemia problem-solving ability in patients with diabetes who had experienced hypoglycaemia in the past six months. In addition, this study aimed to identify predictors associated with higher problem-solving ability scores.

## Methods

### Study design

A cross-sectional study using a self-administered questionnaire was conducted between October 2017 and May 2018 in three Arab countries (Jordan, Saudi Arabia and Kuwait).

### Sampling strategy

A convenience sample of all eligible patients was invited to participate in the study from the diabetes clinics in the participating healthcare centres (one diabetes healthcare centre and two hospitals in Jordan, two healthcare centres and one hospital in Saudi Arabia and fourteen healthcare centres and three hospitals in Kuwait). This sampling technique is a type of non-probability sampling in which participants from the target population who meet the inclusion criteria of the study, and who are easily accessible due to geographical proximity, availability at a given time, or they are willing to take part in the study, are included. The severity of hypoglycaemic events ranged from experiencing typical symptoms of hypoglycaemia (e.g. palpitation, tremor and sweating) to requiring assistance from another person and hospitalisation.

The inclusion criteria were: a) patients with DM aged 18 years and above; b) patients using antidiabetic medications treatment, whether oral antidiabetic medications, insulin or both; c) patients who had experienced hypoglycaemic events during the past six months; and d) patients who had no apparent cognitive deficit. Patients were excluded if they were: a) below 18 years of age; b) unable to understand the Arabic language; c) unable to participate in this study due to physical or emotional distress; and d) pregnant women with gestational diabetes.

Recruitment of participants was conducted by the researcher (AN) in Jordan, and by other healthcare professionals and researchers in Saudi Arabia (FT, NB and HA), and in Kuwait (DA, ZA and MA). In the case that a patient was unable to complete the questionnaire by him/herself, the questionnaire was completed with assistance from the researcher.

With assistance from clinic receptionists, the researcher(s) identified eligible patients attending physician appointments, or by asking the patients themselves about any previous history of experiencing hypoglycaemia. Subsequently, prospective participants were approached and invited to participate in the study. For patients who agreed to participate, the questionnaire was administered to them after explaining the study’s aim and objectives. Patient consent forms and information sheets (in Arabic) were provided to the patients for further clarification about the study. In addition, patients were informed that by completing and returning the questionnaire, this would be considered written consent and agreement to participate in the study. If any patients were illiterate and unable to write, the questionnaire was completed with the assistance of the researcher.

### Sample size

Lack of research on hypoglycaemia in Jordan, Saudi Arabia and Kuwait, necessitated that the sample size was based on the original HPSS study [13]. G-power software version 3.1 with a significance level α = 0.05, power = 0.80 and a small effect size of 0.20 was used, providing a total sample size of 870 patients (290 patients from each site).

### Ethical considerations

Approval for this study was obtained from the ethics committees of the participating centres in Jordan (No. 170108/MH 32/112), Saudi Arabia (equivalent approval received) and Kuwait (No. 2017/680). Moreover, ethical approval was obtained for this study from the UCL Research Ethics Committee (Project ID: 7915/002). Permission and approval for the use of the study questionnaire were acquired from the corresponding author of the original study.

### Questionnaire tool

A previously validated questionnaire, the HPSS questionnaire [5], was translated into the Arabic language and used in this study. The HPSS questionnaire was originally developed and validated by Wu and colleagues to evaluate the problem-solving skills of patients with diabetes by assessing their attitudes and perceptions towards their previous hypoglycaemic events [5]. The HPSS questionnaire is composed of two sub-scales (problem orientation and problem-solving skills), covering 24 items assessing patients’ problem-solving abilities regarding seven factors which include problem orientation (problem-solving perception and detection control) and problem-solving skills (identifying problem attributes, setting problem-solving goals, seeking preventive strategies, evaluating strategies and immediate management) [13]. In addition, the following information was collected: patient demographics (age, gender, educational level, marital status and employment status), type of diabetes mellitus, duration of the disease, diabetes therapy and whether the patient had been hospitalised within the past six months due to hypoglycaemia).

The use of a pre-existing questionnaire has the advantage of being a validated and tested instrument, which increases the reliability of its measure [14]. In addition, the adaptation and use of previously created questionnaires allow comparison with different populations [15]. Patients with DM, who participated in the study, were asked about the degree of applicability of each item using a five-point Likert scale. Patients’ responses ranged from 0 to 4, where 0 means “not at all true of me” and 4 means “extremely true of me”. There are six items (items number 1–4 and 23, 24) that were negatively worded and thus were reversely scored during the analysis, where 0 means “extremely true of me” and 4 means “not at all true of me”. The total possible score for the HPSS questionnaire ranged between 0 and 96 [13] and can be interpreted based on the mid-point of the highest possible score of the scale (equal to 48). The higher the score, the better the hypoglycaemia problem-solving ability [13].

### Translation and adaptation of the HPSS questionnaire

Arabic is the official language and the most commonly used language among the general population in the Arab countries within the Middle East. This questionnaire instrument was therefore translated into the Arabic language (with no specific dialect) so it could be easily understood and completed by all Arabic-speaking patients.

The forward and backward translation technique was used for the translation of this questionnaire. This translation technique consists of three main stages: a) forward translation; b) expert panel assessment; and c) backward translation [16].

The forward translation was completed by two pharmacists (AJ and EG) whose first language was Arabic and had a proficient level of English. The process involved an independent translation of the English version of the HPSS questionnaire into the Arabic language.

The two translators focused on the conceptual translation rather than the literal (word-for-word translation) when translating the original questionnaire into the Arabic language. In addition, they used standard Arabic language (with no specific dialect) that could be understood by all Arabic language speakers. Moreover, they translated the items of the questionnaire without any technical or vernacular terms to make it more comprehensible to the targeted population.

As recommended by the WHO [16], an expert panel consisting of two bilingual physicians (AR and AN), who spoke Arabic and English, and the two original translators (AJ and EG) were required to revise and assess the first draft of the Arabic version of the HPSS questionnaire by making cultural adaptations, providing suggestions for any rewording and assessing the practicality of its use. They were provided with the Arabic version of the questionnaire, the original English HPSS questionnaire and an adapted assessment tool, which contained specific criteria related to questionnaire translation validation [17]. The Arabic version was satisfactory and reflected the same concepts covered by the original HPSS English questionnaire.

The final Arabic version that was drafted through the forward translation and the assessment of the expert panel was followed by a backward translation. This involved the backward translation of the produced Arabic version into the English language. This step was undertaken by a bilingual pharmacist (AG) who had no prior knowledge of the objectives of the study or of the original questionnaire. Finally, the back-translated draft of the questionnaire was compared to the original English language version to assess if they were conceptually equivalent. This assessment was undertaken by a pharmacist (MB) whose first language is English. No changes were suggested by the pharmacist. The two drafts were comparable.

### Pre-testing of the Arabic version of the questionnaire

The Arabic version of the HPSS (see S1 Table) was checked for clarity and comprehensibility by five healthcare professionals from three Arab countries and they confirmed that, based on their experience, it would be easily understood by patients.

A pilot study using the Arabic version of the HPSS self-administered questionnaire was then conducted on 15 patients in Jordan, who met the inclusion criteria for the study. Patients were asked about the clarity and comprehensibility of the questionnaire, and if any of the questions were difficult to understand. In addition, patients were asked if any of the questions were considered unacceptable or offensive. Patients confirmed that the questionnaire was considered easy to understand and to complete.

### Reliability and consistency of the questionnaire

The Cronbach’s alpha measures for the seven factors from the original HPSS questionnaire ranged between 0.70 and 0.86, and the overall Cronbach’s alpha measure for the HPSS was 0.83. The overall test-retest reliability was 0.81; this was undertaken by re-administrating the questionnaire to the targeted patients after two to four weeks. This identified the HPSS questionnaire as having a good to excellent stability.

### Data collection

A total of 895 patients with DM participated in the study (Jordan = 300, Saudi Arabia = 302, and Kuwait = 293). In Jordan, the recruitment of participants was conducted in three healthcare centres in Amman. In Saudi Arabia and Kuwait, it was conducted in three healthcare centres in Mecca in Saudi Arabia, and seventeen healthcare centres in four governorates in Kuwait.

### Statistical analysis

Data were analysed using the SPSS software, version 22. The descriptive analysis was reported as mean (± standard deviation [SD]) for normally distributed quantitative variables and as median (interquartile range [IQR]) for non-normally distributed quantitative variables. Kolmogorov Simonov and Shapiro Wilk tests were used to check the normality of the data. Categorical data were reported as percentages and frequencies. Patients' scores were interpreted as a continuous scale based on the scale midpoint, where scores above the midpoint identified stronger problem-solving ability for that factor.

The Mann-Whitney U test/Kruskal-Wallis test and Spearman’s correlation coefficient were used to compare the median scores between different demographic groups and to analyse the correlation between continuous independent variables and patients’ HPSS scores. Fisher’s least significance difference (LSD) post hoc test was conducted to identify the source of significate variation within each group. In addition, significant predictors of hypoglycaemia problem-solving abilities were determined using multiple linear regression analysis after applying log-transformation for the data. A confidence interval of 95% (p < 0.05) was applied to represent the statistical significance of the results and the level of significance was assigned as 5%.

## Results

### Patients’ characteristics

A total of 895 patients with DM participated in the study (Jordan = 300, Saudi Arabia = 302, and Kuwait = 293). Table 1 details the baseline characteristics of the patients in the three countries. The majority of patients (n = 469, 52.4%) were males, married (n = 757, 84.6%), educated (n = 848, 94.7%) and unemployed or retired (n = 553, 61.8%). Type 2 DM was the most common type of diabetes (n = 786, 87.8%), and most of the patients used combination therapy of oral antidiabetic medications and insulin (n = 321, 35.9%). The average age of the patients was 53.5 years (SD = 13.7), with a mean duration of diabetes of 12.2 years (SD = 8.6). There were 93 patients (10.4%) with a previous history of severe hypoglycaemia that had led to hospital admission during the past six months.

**Table 1:** Patient characteristics from each country.

Demographics	Overall (n = 895)	Jordan (n = 300)	Saudi Arabia (n = 302)	Kuwait (n = 293)
**Gender** No. (%)				
Male	469 (52.4)	183 (61.0)	160 (53.0)	126 (43.0)
**Age (years; mean and SD)** No. (%)	53.5 ± 13.7	60.0 ± 9.4	50.1 ± 13.2	50.3 ± 15.4
**Marital status** No. (%)				
Married	757 (84.6)	294 (98.0)	242 (80.1)	221 (75.4)
**Educational level** No. (%)				
Not educated	47 (5.3)	18 (6.0)	13 (4.3)	16 (5.5)
Completed primary or lower	188 (21.0)	127 (42.3)	33 (10.9)	28 (9.6)
Completed secondary grade	257 (28.7)	67 (22.4)	117 (38.7)	73 (24.9)
College/university or above	403 (45.0)	88 (29.3)	139 (46.0)	176 (60.1)
**Employment status** No. (%)				
Unemployed or retired	553 (61.8)	261 (87.0)	141 (46.7)	151 (51.5)
Employed	342 (38.2)	39 (13.0)	161 (53.3)	142 (48.5)
**Type of diabetes mellitus** No. (%)				
Type 1 diabetes mellitus	109 (12.2)	11 (3.7)	31 (10.3)	67 (22.9)
Type 2 diabetes mellitus	786 (87.8)	289 (96.3)	271 (89.7)	226 (77.1)
**Duration of the disease** No. (%)				
Less than 5 years.	235 (26.3)	73 (24.3)	109 (36.1)	53 (18.1)
Between 6 and 10 years	223 (24.9)	75 (25.0)	73 (24.2)	75 (25.6)
Between 11 and 15 years	175 (19.6)	63 (21.0)	57 (18.9)	55 (18.8)
More than 15 years	262 (29.3)	89 (29.7)	63 (20.9)	110 (37.5)
**Diabetes medication regimen** No. (%)				
Oral antidiabetic medications	316 (35.3)	166 (55.3)	45 (14.9)	105 (35.8)
Insulin	258 (28.8)	56 (18.7)	114 (37.7)	88 (30.0)
Both (Oral medications and insulin)	321 (35.9)	78 (26.0)	143 (47.4)	100 (34.1)
**Hospital admission for hypoglycaemic episodes in the previous 6-months** No. (%)				
Yes	93 (10.4)	17 (5.7)	10 (3.3)	66 (22.5)

### Patients’ problem-solving ability

Table 2 shows the median scores for hypoglycaemic problem-solving ability. Total hypoglycaemia problem-solving ability scores of the patients ranged from 2 to 92, with a median score of 63.00 (IQR = 13.00). Patients had a higher problem-solving skill score with 68.1% compared to their problem-orientation score, 58.3%. Patients had the highest sub-scale scores (75.0%) for detection control, setting problem-solving goals, and evaluating strategies, whereas the lowest sub-scale scores (50.0%) were for problem-solving perception and immediate management.

**Table 2:** Participant median scores for hypoglycaemia problem-solving ability (n = 895).

			Score/ Scale		Patients score out of 100%
Scale/Subscale	Number of Item	Range	Median	IQR	
**Hypoglycaemia Problem-Solving Scale**	24	0–96	63.00	13.00	65.6
**Problem orientation**	6	0–24	14.00	7.00	58.3
Problem-solving perception	4	0–16	8.00	6.00	50.0
Detection control	2	0–8	6.00	2.00	75.0
**Problem-solving skill**	18	0–72	49.00	13.00	68.1
Identifying problem attributes	5	0–20	14.00	5.00	70.0
Setting problem-solving goals	3	0–12	9.00	3.00	75.0
Seeking preventive strategies	4	0–16	11.00	6.00	68.8
Evaluating strategies	4	0–16	12.00	4.00	75.0
Immediate management	2	0–8	4.00	3.00	50.0

Abbreviation: IQR, Interquartile Range

### Patient demographics and hypoglycaemic problem-solving ability

Table 3 presents patient demographics data and their hypoglycaemia problem-solving ability scores.

**Table 3:** Hypoglycaemia problem-solving ability score by patient characteristics (n = 895).

	HPSS score		
Variable	Median	IQR	P-value	
**Country**				
Jordan	66.00	9.00	0.000***	
Saudi Arabia	63.00	10.00		
Kuwait	52.00	24.50		
**Gender**				
Males	64.00	12.00	0.015*	
Females	62.00	16.00		
**Age**				
18–39 years	61.00	24.00	0.233	
40–59 years	63.00	14.00		
60 years and above	63.00	11.00		
**Marital status**				
Unmarried	60.00	24.00	0.052	
Married	63.00	13.00		
**Educational level**				
Illiterate	57.00	17.00	0.008**	
Completed primary or lower	61.00	11.00		
Completed secondary grade	63.00	14.00		
College/university or above	64.00	16.00		
**Employment status**				
Unemployed or retired	62.00	12.50	0.274	
Employed	64.00	17.00		
**Type of diabetes mellitus**				
Type 1 diabetes mellitus	55.00	34.00	0.078	
Type 2 diabetes mellitus	63.00	12.00		
**Duration of the disease**				
Less than 5 years	63.00	13.00	0.994	
Between 6 and 10 years	63.00	15.00		
Between 11 and 15 years	63.00	14.00		
More than 15 years	62.00	16.00		
**Diabetes medication regimen**				
Oral antidiabetic medications	62.00	14.00	0.360	
Insulin	63.50	19.00		
Both (Oral medications and insulin)	63.00	12.00		
**Hospital admission for hypoglycaemic episodes in the previous 6-months**				
No	63.00	13.00	0.001***	
Yes	56.00	17.50		

*p < 0.05

**p < 0.01

***p < 0.001

Abbreviation: IQR, Interquartile Range

Participants’ HPSS scores significantly differed by country, age, gender, marital status, educational level, type of diabetes mellitus, and whether or not they had been admitted to hospital for hypoglycaemia during the past six months (p < 0.05). The problem orientation sub-scale score showed statistically significant differences between patients based on their gender only (p < 0.05). Problem-solving skills scores differed significantly according to patients’ gender, age, marital status, educational level, employment status, type of diabetes, type of treatment regimen and having been admitted for hypoglycaemia during the past six months (p < 0.05). The three countries contributed to the significant difference and Kuwait had the most significant contribution in this variation. Regarding the education variable, the LSD test confirmed that “completed secondary grade” was the main source of significant variation between groups.

Multiple linear regression analysis showed that married patients and those who had a higher educational level had greater problem-solving ability scores than patients with lower educational level, non-educated patients and unmarried patients (p < 0.05) Table 4.

**Table 4:** Multiple regression analysis predicting hypoglycaemia problem-solving ability.

	Model 1^a^									Model 2^b^		
Variable	B			SE			ß			B	SE	ß
Demographic data												
**Age (years)**	0.150			0.040			0.157***			0.077	0.044	0.080
**Males**	1.648			0.906			0.063			1.555	0.899	0.060
Married	3.252			1.332			0.090**			2.687	1.358	0.074*
Educational level												
**Completed primary or lower**	3.917			2.152			0.122			3.498	2.135	0.109
**Completed secondary grade**	5.687			2.140			0.197**			5.051	2.129	0.175*
**College/university or above**	5.597			2.149			0.213**			5.450	2.133	0.208*
Employed	-0.028			1.044			-0.001			-0.525	1.044	-0.020
Type of diabetes mellitus												
**Type 1 diabetes mellitus**										-6.686	1.861	-0.168***
Diabetes medication regimen												
**Prescribed insulin**										3.438	1.244	0.119**
**Prescribed both (oral medications and insulin)**										1.925	1.021	0.071
Previously admitted for hypoglycaemia										-4.308	1.404	-0.101**
Constant	43.500		3.041							41.369	3.204	
Adjusted R^2^								0.045				0.068
P-value								0.000				0.000

*p < 0.05

**p < 0.01

***p < 0.001

a: includes age, gender, marital status, educational level, and employment status

b: includes age, gender, marital status, educational level, employment status, type of diabetes, diabetes medicaiton regimen, and previous history with hypoglycaemia admission

B: the average change in the depenedent variable associated with a 1 unit change in the independent variable, statisitcally controlling for the other independent variables; SE: it is the standard deviation of its sampling distribution or an estimate of that standard deviation; ß: a statistical measure that compares the strength of the effect of each individual independent variable to the dependent variable

## Discussion

This study raises awareness about the effect of patients’ psychological factors (perception of their health problem) on their problem-solving abilities towards hypoglycaemic events of DM. The findings of our study highlight the importance of educating patients about their disease, and thus enabling them to apply preventive and proactive strategies to decrease the re-occurrence of unwanted hypoglycaemic events.

To be able to compare the results of the current study to a study conducted in Taiwan which used the same questionnaire [13] we presented the mean score (SD) per item. This allows for direct comparison. Compared to that study, our study observed a higher overall mean problem-solving ability score (60.09 [SD = 13.05] Table 2 versus 58.22 [SD = 17.98]), and higher mean problem-solving skill sub-scale score (46.53 [SD = 11.55] Table 2 versus 40.32 [SD = 16.84]). A lower mean score for the problem orientation sub-scale was identified (13.56 [SD = 4.68] Table 2 versus 17.90 [SD = 4.18]), which could be due to differences in culture, level of education and access to healthcare services between patients from different countries [18–20].

In contrast to the study in Taiwan, the current study showed a higher mean problem-solving skill score per item (2.59 [SD = 0.64] Table 2 versus 2.24 [SD = 0.94]) compared to the problem orientation score of (2.26 [SD = 0.78] Table 2 versus 2.98 [SD = 0.70]) (p < 0.05) among the participants. This means that the patients had a negative attitude towards managing their hypoglycaemic events and had negative perceptions, with minimum engagement, regarding solving their hypoglycaemia [21]. A lower problem orientation score reflects a weakness in patients’ ability and involvement in assessing their health problems, and consequently difficulties in solving them, resulting in little proactive management for their health problem [22–25]. Therefore, patients view their health problem with unclear understanding and direction on how to manage it [26,27].

Lacking awareness about hypoglycaemia and how to manage it has been reported among different eastern Mediterranean countries and in the UK [28, 29]. A study by Tanjia et al. in 2016 also identified that a substantial proportion (40%) of patients with diabetes lack knowledge about hypoglycaemia symptoms and how to manage them. Poor perceptions about hypoglycaemia can lead to hazardous practices undertaken by patients, for example, ignoring hypoglycaemia once it has been detected or going to sleep [30, 31]. In a study in western Pennsylvania, it was reported that patients who frequently self-monitored their blood glucose levels (SMBG) were better able to resolve hypoglycaemic events [9]. However, it was affirmed in that study that the patients needed more training on how to interpret the results of SMBG to make appropriate lifestyle and/or therapeutic changes after detecting the event. On the other hand, other studies have reported that patients with DM were aware of diabetes symptoms and complications, severity of their events and the actions required to manage them. In those studies, patients were not only able to adjust their lifestyle based on their metabolic needs but they also adapted their daily activities, work schedules and personal relationships to help prevent hypoglycaemic events [32–34]. Different factors should be taken into consideration when dealing with patients with DM who are at higher risk of developing diabetes complications. A recent study in the Islamic Republic of Iran has emphasised the role of positive family history of T2DM, controlled hypertension and residence area and their relation with DM and its control [35].

To better understand the effect of impaired awareness on the health-related actions taken by patients, it is crucial to interpret it from the concept of health behavioural models such as the health belief model, which is a function of patients’ beliefs about perceived threats and an evaluation of the value (benefits) of taking a health action weighed against its psychological costs (barriers) [36]. Perceived threat is split into two dimensions, perceived susceptibility and perceived severity. Perceived susceptibility indicates that an individual must believe that they are susceptible to the problem (e.g. hypoglycaemia). Perceived severity indicates that an individual should understand the seriousness of the problem (e.g. hypoglycaemia) [37]. For example, if patients believe that hypoglycaemia is serious, that they are vulnerable to it, that following medical recommendations will reduce threats, that their health actions are effective and that the benefits of taking proper actions outweigh the risks of hypoglycaemia, they will be more likely to undertake recommended health-related actions, e.g. improving their perceptions and attitudes towards hypoglycaemia.

Confirming the findings of a previous study in Taiwan [13], we found that having a higher educational level was statistically significantly associated with higher hypoglycaemia problem-solving abilities. Having a higher educational level could result in developing patients’ problem-solving abilities and having a greater understanding of health problems and their effect on long-term conditions and mortality, and thus help patients to adopt a healthier lifestyle and preventive strategies [8,38–43]. Furthermore, previous studies have reported that higher level of [39] education among family members was also associated with better adoption of medical information advice to resolve any health problems for their relatives [44, 45]. On the other hand, conflicting data have been revealed from a study in Australia [46] which found that higher educational attainment was associated with severe hypoglycaemia in patients with type 2 diabetes. However, this was related to the fact that educated patients might be more aware of the risks of poor glycaemic control, which increases their access to healthcare services and makes them more engaged in self-management practices and consequently makes them more vulnerable to hypoglycaemia.

Patients’ employment status has been reported to be related to patients’ behaviour because unemployment is negatively associated with patients’ health outcomes, making them more depressed, mentally unstable, and more likely to have a poorer perception of their health status [47–50]. However, in this study employment status did not reflect a statistically significant difference between the patients (p > 0.05). This may be because in our study most of the patients were older (mean age 53.5 years ± 13.7) and retired, not unemployed, which reflects a better socio-economic status, and thus better health conditions compared to patients who are unemployed.

This study also identified that being older, married, prescribed insulin and not being admitted to hospital for hypoglycaemia during the past six months were predictors of a higher problem-solving ability score (p < 0.05). In contrast to the theory suggested by the Social Problem Solving Inventory-Revised, where social problem-solving ability decreases with older age [51], in our study being older and prescribed insulin were positively associated with more problem-solving abilities. This could be because older patients who are on insulin therapy receive more instructions and precautionary advice from their treating physicians because it is a high-risk medication associated with hypoglycaemia, and thus they know more about their expected hypoglycaemic event and can manage it better. Additionally, patients’ concerns about experiencing recurrent hypoglycaemic events might motivate them to find out more about diabetes and related events, which would increase their ability to self-manage their condition, and thus decrease the probability of experiencing unwanted events in the future. Unlike the study by Wu et al., this study did not find a statistically significant association between having T1DM and having a higher problem-solving ability score [13]. Some studies have identified that insulin treatment and its longer duration of use are predictors of first and multiple hypoglycaemic episodes [46, 52]. Thus, insulin treatment rather than type of diabetes seems to be the important predictor of hypoglycaemic events.

A study by Davis and colleagues suggested that a history of previous hospitalisation for severe hypoglycaemia was a strong predictor of the subsequent events [46]. This might reflect the fact that patients with poor awareness of severe hypoglycaemia are more susceptible to its subsequent occurrence. Patients who experience severe or frequent hypoglycaemic episodes have lower general health, lower quality of life and greater fear of hypoglycaemia [53, 54]. This might reflect similar findings to the current study, were patients without a history of frequent hypoglycaemia had more problem-solving abilities than those with a history of more frequent episodes. Conflicting data have been reported by Leiter et al., where patients found to be able to imply modifications to their insulin doses and lifestyle changes post a severe hypoglycaemic episode [55]. Finally, the findings of this study emphasise the importance of these problem-solving abilities and filling the gap in patients with DM in the four components of the Hill-Briggs problem-solving model: problem-solving skill, problem-solving orientation, disease-specific knowledge and transfer of past experience [56].

### Strengths and limitations

This study has several strengths. This is the first study in Middle Eastern Arabic-speaking countries to investigate patients’ attitudes towards and perceptions of their previous hypoglycaemic events. The study population included patients with T1DM and T2DM, which increases the generalisability of these findings. The study questionnaire was distributed in three Arabic-speaking countries in the Middle East, which widens the scope of the generalisability of our findings to Arab countries in the Middle East. Additionally, the translation of the instrument using forward and backward methods based on conceptual understanding was another strength of the study.

However, there are some limitations. The study design itself, a cross-sectional survey design, limited our ability to identify causality between study variables. No prior study had been conducted in the Middle East using the HPSS, which prevented us from comparing our findings with Arabic-speaking countries of a similar healthcare environment and culture. It was not possible to collect other information about the severity of the disease such as the presence of comorbidities and the presence of polypharmacy. The piloting of the translated questionnaire was conducted in one country (Jordan). This led to a difficulty for a few patients (in Kuwait) in understanding a few terms due to a minor difference in the dialect. However, since the researchers were available during the questionnaire completion, they were able to provide any explanation needed for these patients. In addition, it was not possible to investigate cultural, religious and economic differences between the three countries in this study as we were restricted by the validity of the questionnaire. However, this could be an important gap on the literature to be addressed in future work. One item in the questionnaire showed an unfavourable Cronbach's alpha value, which was the immediate management sub-scale. This could be due to the small number of items per variable, which could have underestimated its internal reliability [57]. Some of the demographic information was merged and presented in two groups only (marital status and employment status), which might restrict our ability to investigate the effect of each sub-category on the risk of hypoglycaemia, as these sub-categories could contribute to differences in the development of health conditions. Finally, we were not able to estimate the response rate for our questionnaire study, which might lead to nonresponse bias, as we could not demonstrate how well the sample drawn from the population of interest, therefore, the findings should be interpreted carefully.

### Implications for practice

The findings of this study suggest that patients with DM with a previous history of hypoglycaemic events, and who are using intensive antidiabetic therapy with a high risk of developing hypoglycaemia (such as insulin and insulin secretagogues), should receive educational programmes that focus on developing their ability to recognise, analyse and solve their health problems [38–40]. Educational interventions could also focus on improving patients’ awareness of the importance of lifestyle. It has been shown that patients with DM, who are at risk of hypoglycaemia due to their antidiabetic medications, could reduce their risk of recurrent episodes by applying some lifestyle modifications such as having an appropriate dietary intake and doing a moderate level of exercise [8, 41, 42].

Understanding the psychological barriers to self-care in patients with DM is also of paramount value. An integrated psychological approach is required for promoting self-care practices among patients to improve health outcomes [58]. Many clinicians depend on educational programmes that provide patients with important information regarding disease management and adherence to self-care behaviours, which could be insufficient for effective diabetes management [59]. Rather clinicians should adapt a problem-solving training approach that is patient-oriented to help patients become more expert in self-management by relating the events to their past experiences [60]. Problem-solving interventions could result in significant improvement in HbA1c as a consequence of behavioural improvement [8, 56, 61]. Finally, problem-solving interventions should extend to include home-based programmes using telephone and telehealth applications in order to overcome barriers to participation such as difficulties in transportation [62]. Involving family, friends and the work environment could also have a positive impact on the patients’ diabetes management and well-being [34].

## Conclusion

Patients’ educational level, marital status, not being admitted to hospital for hypoglycaemia during the past six months, and being prescribed insulin were significant predictors of high problem-solving ability. This highlights the importance of focusing on providing patients with education to increase their knowledge of diabetes and their ability to identify and self-manage hypoglycaemic events.

## Supporting information

S1 Table The Arabic translated questionnaire. (DOCX)
