# Supplementary material for: Attitudes and perceptions towards hypoglycaemia in patients with diabetes mellitus: A multinational cross-sectional study
Source: PLoS One. 2019 Oct 24;14(10):e0222275. doi: 10.1371/journal.pone.0222275 (PMC6812873; doi:10.1371/journal.pone.0222275)
Supplement: S1 Table — (DOCX) [file pone.0222275.s001.docx]

**Table S1: The Arabic translated questionnaire**

**عزيزي المريض / عزيزتي المريضة**

**يرجى منك أن تشاركنا رأيك بشأن مواقفك وتصوراتك تجاه مشكلة نقص السكر الدم.**

**هذا الاستبيان يهدف لفهم مواقف وتصورات مرضى السكري تجاه مشكلة نقص السكر في الدم لمعرفة قدرتهم على حل مشكلة نقص السكر في الدم . لا يوجد إجابة صحيحة أو خاطئة. يستغرق استكمال الاستبيان من ١٠-١٥ دقيقة.**

**مشاركتك قيمة بالنسبة لنا، فهي ستساعد في تطوير الخدمات الصحية المقدمة لمرضى السكري.**

**ستعامل معلوماتك بسرية تامة ولن يستطيع أحد التعرف علي هويتك. لن نسأل عن اسمك في أي مكان بالإستبيان.**

**المعلومات المجمعة سوف تستخدم لغرض الدراسة فقط.**

**إذا كان لديك أي سؤال حول الإستبيان، لا تتردد بالاتصال بي على الإيميل الخاص بي.**

**نشكر لكم حُسن تعاونكم**

**الصيدلاني عبدالله نصر: طالب دكتوراة في الصيدلة السريرية و إقتصاديات الدواء**

**مشرفي البحث:**

**دكتورة لي وي**

**بروفيسور اين ونج**

جامعة كلية لندن

*** يعتبر استكمال الاستبيان وإعادته موافقة خطية واتفاقاً على المشاركة في البحث.**

**** المشارکة طوعیة ولديك حریة الانسحاب من البحث في أي وقت و دون إبداء أي سبب.**

***** الرجاء اختيار خيار واحد فقط وهو الأكثر ملائمة لك.**

**العُمر (كتابة): (....................)**

☐18 - 39 سنه

☐40 - 59 سنة

☐ أكثر من 60 سنة

**الجنس**

☐ ذكر

☐ أنثى

**الحالة الإجتماعية:**

☐ غير متزوج (ة)

☐ متزوج (ة)

**المستوى التعليمي:**

☐ غير متعلم.

☐ أنهى المرحلة الإبتدائية أو أقل منها.

☐أنهى المرحلة الثانوية أو اقل منها.

☐ مستوى كلية أو جامعي أو دراسات عليا.

**الحالة الوظيفية:**

☐لا يعمل.

☐ يعمل.

**نوع مرض السكري المصاب به المريض:**

☐سكري من النوع الأول “أصاب المريض قبل سن الثامنة عشر”

☐ سكري من النوع الثاني” أصاب المريض بعد سن الثامنة عشر”

**منذ متى يعاني المريض من مرض السكري:كتابة (....................)**

☐أقل من 5 سنوات.

☐ من 5 إلى 10 سنوات.

☐ من 10 إلى 15 سنة.

☐ أكثر من 15 سنة.

**نوع علاج السكري الذي يتلقاه المريض:**

☐ حبوب لخفض مستوى السكر في الدم.

☐ حقن أنسولين.

☐ كلاهما (حبوب و حقن أنسولين).

**هل زار المريض أحد المستشفيات كحالة طارئة بسبب حدوث نقص السكر في الدم خلال ال6 شهور الماضية.**

☐لا.

☐ نعم.

| **غير صحيح على الإطلاق بالنسبة لي** | **صحيح قليلاً بالنسبة لي** | **صحيح باعتدال بالنسبة لي** | **صحيح جداً بالنسبة لي** | **صحيح للغاية بالنسبة لي** |  |  |
| --- | --- | --- | --- | --- | --- | --- |
|  |  |  |  |  | **عندما تفشل محاولتي في منع نقص السكر في الدم ، أصبح محبط ولا أستطيع التفكير بشكل واضح.** | **1** |
|  |  |  |  |  | **الصعوبة التي أواجهها في منع نقص السكر في الدم تجعلني أشعر بالاكتئاب أو الغضب.** | **2** |
|  |  |  |  |  | **أنا غالباً ما ينتابني القلق تجاه كيفية منع نقص السكر في الدم لكنني لا أتخذ أي إجراء لمعالجة ذلك.** | **3** |
|  |  |  |  |  | **عندما لا أستطيع منع نقص السكر في الدم أشعر بالحُمق.** | **4** |
|  |  |  |  |  | **أنا أعرف كيف أسيطر على نقص السكر في الدم.** | **5** |
|  |  |  |  |  | **أنا لا أستسلم عندما تفشل محاولتي الأولية لمنع نقص السكر في الدم بشكل فعال، وأنا على يقين أنني سوف أجد الحل المناسب لها.** | **6** |
|  |  |  |  |  | **عندما يحدث نقص سكر الدم، أقوم بفحص أي شيء قد يساهم بحدوث نقص سكر الدم.** | **7** |
|  |  |  |  |  | **عندما تكون جهودي لمنع نقص السكر في الدم غير فعالة، أعود للأخطاء التي ارتكبتها وأحاول بطرق أخرى.** | **8** |
|  |  |  |  |  | **عندما أكون غير راضياً عن نتائج منع نقص السكر في الدم، سوف أجد طريقة أفضل وأحاول مرة أخرى.** | **9** |
|  |  |  |  |  | **عندما تفشل محاولتي في منع نقص السكر في الدم ، سوف أحلل و أحدد خطئي.** | **10** |
|  |  |  |  |  | **لمنع نقص السكر في الدم، أحاول قدر الإمكان أن أتعلم المزيد من المعلومات حول حدوث نقص السكر في الدم.** | **11** |
|  |  |  |  |  | **عندما أحاول السيطرة على نقص السكر في الدم، أتذكر جميع الأهداف التي وضعتها.** | **12** |
|  |  |  |  |  | **عندما أحاول منع نقص السكر في الدم، أضع هدفاً لأعرف ما أحتاج تحقيقه.** | **13** |
|  |  |  |  |  | **سأحاول منع نقص السكر في الدم وتحقيق جميع الأهداف التي وضعتها.** | **14** |
|  |  |  |  |  | **أنا عادة أتحدث مع عائلتي عندما أحاول منع نقص السكر في الدم.** | **15** |
|  |  |  |  |  | **أنا أتحدث مع المتخصصين في الرعاية الصحية عندما يصبح منع نقص السكر في الدم معقداً و صعباً.** | **16** |
|  |  |  |  |  | **عندما يصبح منع نقص السكر في الدم معقداً و صعباً ، أطلب المساعده من أصدقائي أو أراقب ما يحصل لجسمي من تغيرات جسدية.** | **17** |
|  |  |  |  |  | **عندما يصبح منع نقص السكر في الدم معقداً و صعباً ، أتعلم كيفية منع نقص السكر في الدم من الناس الذين يعانون من نفس مشكلتي.** | **18** |
|  |  |  |  |  | **بعد تنفيذ طريقة منع نقص السكر في الدم، أقوم بتقييم فعاليتها.** | **19** |
|  |  |  |  |  | **عندما أحاول منع نقص السكر في الدم، أحاول بطريقتي الخاصة لزيادة فرصة النجاح.** | **20** |
|  |  |  |  |  | **عندما أحدد أفضل طريقة لمنع نقص السكر في الدم، أحاول أن أتوقع النتيجة المحتملة.** | **21** |
|  |  |  |  |  | **أنا أفهم أن منع نقص السكر في الدم هي واحدة من المشاكل التي يجب حلها في مجال رعاية مرض السكري.** | **22** |
|  |  |  |  |  | **عندما أعاني من نقص السكر في الدم، عادة ما أتناول وجبة خفيفة، و أوقف جميع النشاطات أو أوقف حقن الأنسولين، ولا أفكر في الوقاية.** | **23** |
|  |  |  |  |  | **بالنسبة لي، نقص السكر في الدم هي مشكلة يمكن التحكم فيها بسهولة ولا تحتاج لاهتمام كبير.** | **24** |

**The original questionnaire**

Dear Patient,

It is kindly requested that you share with us your opinions regarding your attitudes and perceptions towards your previous hypoglycaemic events.

This questionnaire aims to understand diabetic patients’ attitudes and perceptions towards their hypoglycaemic events to estimate their problem-solving ability towards hypoglycaemia. There is no right or wrong answer. The questionnaire should not take more than 10-15 minutes to be completed. Your contribution is greatly valued, as it will help towards improving health care for patients with diabetes mellitus.

The questionnaire is completely anonymous and the researcher(s) will not be able to identify you from the responses provided. You will not be asked for your name anywhere on the questionnaire. We will use the collected information for research purposes only.

If you have any questions about this questionnaire, please do not hesitate to contact me at the email address below.

Thank you for your co-operation.

Abdallah Y Naser

PhD student in Clinical Pharmacy and Pharmacoeconomics.

Email: [abdallah.naser.15@ucl.ac.uk](mailto:abdallah.naser.15@ucl.ac.uk).

Supervisors:

Dr. Li Wei

Professor. Ian Wong

University College London

**Notes:**

***** Completing and returning the questionnaire is considered as written consent and agreement of participation.

****** Participation is voluntary and you are free to withdraw from the research at any time without giving any reason.

******* Please choose only one choice, which is the most applicable one to you.

**1. Age (…………….)**

☐ 18–39 years

☐ 40–59 years

☐ More than 60 years

**2. Gender**

☐ Males

☐ Females

**3. Marital status**

☐ Unmarried

☐ Married

**4. Educational level**

☐ Not educated

☐ Completed primary or lower

☐ Completed secondary grade

☐ College/university or above

**5. Employment status**

☐ Unemployed

☐ Employed

**6. Type of diabetes mellitus**

☐ Type 1

☐ Type 2

**7. Duration of the disease (……………..)**

☐ Less than 5 years.

☐ Between 5 and 10 years.

☐ Between 10 and 15 years.

☐ More than 15 years.

**8.Diabetes medication regimen**

☐ Oral

☐ Insulin

☐ Oral medication and insulin

**9.Hospital admission for hypoglycaemic episodes in the previous 6-months**

☐ No

☐ Yes

|  | Not at all true of me | Slightly true of me | Moderately true of me | Very true of me | Extremely true of me |
| --- | --- | --- | --- | --- | --- |
| **1.** When my attempt to prevent hypoglycaemia fails, I become discouraged and cannot think clearly. |  |  |  |  |  |
| **2.** The difficulty I encounter in preventing hypoglycaemia makes me feel depressed or angry. |  |  |  |  |  |
| **3.** I often worry about how to prevent hypoglycaemia but have not taken any action to address it. |  |  |  |  |  |
| 4. When I cannot prevent hypoglycaemia, I feel stupid. |  |  |  |  |  |
| **5.** I know how to handle hypoglycaemia. |  |  |  |  |  |
| **6.** I do not give up when my initial attempt to effectively prevent hypoglycaemia fails, and I believe that I will ultimately find the best approach to solve it. |  |  |  |  |  |
| **7.** When hypoglycaemia occurs, I examine for any event that may contribute to the occurrence of hypoglycaemia. |  |  |  |  |  |
| **8.** When my efforts to prevent hypoglycaemia are ineffective, I return to where I made the mistakes and attempt other methods. |  |  |  |  |  |
| **9.** When I am not satisfied with the results of preventing hypoglycaemia, I will find a better method and attempt it again. |  |  |  |  |  |
| **10.** When my attempt to prevent hypoglycaemia fails, I will analyse and identify my mistake. |  |  |  |  |  |
| **11.** To prevent hypoglycaemia, I attempt to learn as much information on the occurrence of hypoglycaemia as possible. |  |  |  |  |  |
| **12.** When I attempt to manage hypoglycaemia, I remember all the goals that I have set. |  |  |  |  |  |
| **13.** When attempting to prevent hypoglycaemia, I set a goal so that I know what I need to achieve. |  |  |  |  |  |
| **14.** I will attempt to prevent hypoglycaemia and achieve all the goals I have set. |  |  |  |  |  |
| **15.** I usually speak with my family when I am attempting to prevent hypoglycaemia. |  |  |  |  |  |
| **16.** I speak with health professionals when hypoglycaemia prevention becomes complex and difficult. |  |  |  |  |  |
| **17.** When hypoglycaemia prevention becomes complex and difficult, I seek help from friends or pay close attention to my physical changes. |  |  |  |  |  |
| **18.** When hypoglycaemia prevention becomes complex and difficult, I learn how to prevent hypoglycaemia from people who have the same problem as mine. |  |  |  |  |  |
| **19.** After implementing the method for hypoglycaemia prevention, I evaluate the effectiveness of this method in preventing hypoglycaemia. |  |  |  |  |  |
| **20.** When preventing hypoglycaemia, I attempt my own method to increase the chance of success. |  |  |  |  |  |
| **21.** When determining the best hypoglycaemia prevention method, I attempt to predict the possible outcome. |  |  |  |  |  |
| **22.** I understand hypoglycaemia prevention is one of the problems that must be resolved in diabetic care. |  |  |  |  |  |
| **23.** When I experience hypoglycaemia, I usually snack, stop all activity, or stop insulin injections, and do not think about prevention. |  |  |  |  |  |
| **24.** To me, hypoglycaemia is an easily manageable problem and does not need to be a concern. |  |  |  |  |  |
